# Supplementary material for: What do part-time employees in Japanese chain restaurants talk about when dissatisfied? Applying Structural Topic Modeling to employee reviews
Source: PLoS One. 2024 Dec 5;19(12):e0313450. doi: 10.1371/journal.pone.0313450 (PMC11620471; doi:10.1371/journal.pone.0313450)
Supplement: S1 Table — The primary distinction between Models 1 and 2 lies in the proportions of topics, with no significant differences otherwise. (PDF) [file pone.0313450.s001.pdf]

S1 Table Topics and labels of Model 2.

Topic #	Topic Label	Top Words	FREX Words	Topic Proportions
1	Store Manager	店長(store manager), パート(part-time), 社員(employee), 辞める(quit), 店(store)	店長(store manager), 機嫌(mood), 気に入る(like), パート(part-time), パワハラ(power harassment)	8.73%
2	Workplace Relations	店舗(store), 多い(many), 良い(good), 人間関係(human relations), 悪い(bad)	仲(relationship), よる(approach), 客層(clientele), 同士(colleagues), 雰囲気(atmosphere)	8.13%
3	Job Fulfillment	仕事(job), 楽しい(fun), やりがい(challenge), 感じる(feel), 出来る(can do)	やりがい(challenge), 楽しい(fun), 向く(suitable), 感じる(feel), 確か(certain)	7.53%
4	Shift Flexibility	シフト(shift), 入れる(to schedule), 休み(holiday), 希望(wish), 週(week)	シフト(shift), 休み(holiday), 予定(schedule), 希望(wish), 週(week)	7.25%
5	Training Process	教える(teach), 分かる(understand), 研修(training), わかる(understand), 先輩(senior)	丁寧(polite), 教える(teach), 教わる(learn), 研修(training), 初日(first day)	7.15%
6	Student Part-Time Jobs	バイト(part-time job), 高校生(high school student), 優しい(kind), 始める(start), 辞める(quit)	バイト(part-time job), 大学生(college student), 高校生(high school student), 始める(start), 友達(friend)	7.01%
7	Newcomer Integration	辞める(quit), 新人(newcomer), スタッフ(staff), 仕事(job), 職場(workplace)	スタッフ(staff), ベテラン(veteran), オープニング(opening), 言い方(way of speaking), 新人(newcomer)	6.14%
8	Labor Cost Reduction	時給(hourly wage), 休憩(break), 店(store), 上がる(increase), 仕事(job)	人件費(labor cost), ワンオペ(single operation), 削減(cut), 削る(trim), 営業(operation)	5.82%
9	Learning	覚える(remember), メニュー(menu), 多い(many), レジ(register), 大変(difficult)	ドリンク(drink), メニュー(menu), 覚える(remember), カフェ(cafe), フード(food)	4.97%
10	Work Stress	やめる(quit), きつい(hard), 精神的(mental), メンタル(mental), 怒る(angry)	やめる(quit), メンタル(mental), 精神的(mental), きつい(hard), 後悔(regret)	4.78%

S1 Table continued.

Topic #	Topic Label	Top Words	FREX Words	Topic Proportions
11	Work Area Dynamics	キッチン(kitchen), ホール(hall), 忙しい(busy), 料理(cooking), フロア(floor)	キッチン(kitchen), ホール(hall), 料理(cooking), 案内(guide), フロア(floor)	4.77%
12	Company	会社(company), 社員(employee), ダメ(not good), 現場(worksite), 無い(none)	ダメ(not good), 会社(company), 現場(site), カメラ(camera), 有る(exist)	3.90%
13	Customer Interaction	お客様(customer), 客(customer), 店(store), かける(speak), 対応(response)	お客様(customer), 汚い(dirty), かける(speak), 利用(use), 接客業(service industry)	3.85%
14	Digital communication	電話(phone), 連絡(contact), 来る(come), 最悪(worst), 出勤(attendance)	連絡(contact), 電話(phone), メール(email), 送る(send), ビデオ(video)	3.77%
15	Role Assignment	仕事(job), ポジション(position), 洗い場(dishwashing area), 担当(in charge), おば(old lady)	バックヤード(backyard), 洗い場(dishwashing area), 軍艦(gunkan maki), うどん(udon), ネタ(sushi toppings)	3.64%
16	Payroll and Interview	給料(salary), 面接(interview), 書く(write), 制服(uniform), 残業(overtime)	制服(uniform), もらえる(can receive), 靴(shoes), 面接(interview), 定時(regular time)	3.53%
17	Low Wage	時給(hourly wage), 最低賃金(minimum wage), 安い(cheap), 給料(salary), 見合う(suitable)	最低賃金(minimum wage), 賃金(wage), 労働(labor), オーナー(owner), 見合う(suitable)	2.46%
18	Break Time Management	食べる(eat), 休憩(break), 確認(confirm), トイレ(toilet), 忙しい(busy)	食べる(eat), 賄い(meal), トイレ(toilet), 手洗い(hand washing), 摩可(possible)	2.37%
19	Cooperate Employee	社員(employee), 店舗(store), 業務(task), アルバイト(part-time job), 当たり前(usual)	発注(order), 業務(task), 社員(employee), トラブル(trouble), 頻繁(frequent)	2.17%
20	Multinational Corporations Careers	マネージャー(manager), クルー(crew), 店舗(store), トレーナー(trainer), 仕事(job)	クルー(crew), トレーナー(trainer), マネージャー(manager), 信じる(believe), 星(star)	2.02%
