# Supplementary material for: What do part-time employees in Japanese chain restaurants talk about when dissatisfied? Applying Structural Topic Modeling to employee reviews
Source: PLoS One. 2024 Dec 5;19(12):e0313450. doi: 10.1371/journal.pone.0313450 (PMC11620471; doi:10.1371/journal.pone.0313450)
Supplement: Attachment — Submitted filename: Response to reviewers.docx [file pone.0313450.s002.docx]

Rebuttal Letter

Dear Editor

In face of PLOS ONE's decision for the manuscript " What Do Part-Time Employees in Japanese Chain Restaurants Talk About When Dissatisfied? Applying Structural Topic Modeling to Employee Reviews ", we thank the editor’s and reviwer’s comments that has been so constructive to elaborate this new version of the submitted paper. All the comments were considered providing the following changes to the points raised by the academic editor and reviewers:

**Journal Requirements**

1. Please ensure that your manuscript meets PLOS ONE's style requirements, including those for file naming. The PLOS ONE style templates can be found at

https://journals.plos.org/plosone/s/file?id=wjVg/PLOSOne_formatting_sample_main_body.pdf and

https://journals.plos.org/plosone/s/file?id=ba62/PLOSOne_formatting_sample_title_authors_affiliations.pdf

My response:

I have modified the manuscript using the PLOS ONE LaTeX template (<https://journals.plos.org/plosone/s/latex>) and corrected the figures to "Fig" and adjusted the author and affiliation descriptions according to the style requirements provided in the URLs.

2. Thank you for stating the following financial disclosure:

“Japan Society for the Promotion of Science”

Please state what role the funders took in the study.  If the funders had no role, please state: "The funders had no role in study design, data collection and analysis, decision to publish, or preparation of the manuscript."

If this statement is not correct you must amend it as needed.

Please include this amended Role of Funder statement in your cover letter; we will change the online submission form on your behalf.

My response:

I have modified my cover letter and added the following statement: "The funders had no role in study design, analysis, decision to publish, or preparation of the manuscript." I omitted the "data collection" part because I considered the data-providing organization to be relevant to data collection activities

3. Thank you for stating the following in the Acknowledgments Section of your manuscript:

“This work was supported by JSPS KAKENHI Grant Number 23K17003. Ad[1]ditionally, the ”Minhyo Review Dataset” was provided by meisterstudio, Inc. via

IDR Dataset Service of National Institute of Informatics.”

We note that you have provided funding information that is currently declared in your Funding Statement. However, funding information should not appear in the Acknowledgments section or other areas of your manuscript. We will only publish funding information present in the Funding Statement section of the online submission form.

Please remove any funding-related text from the manuscript and let us know how you would like to update your Funding Statement. Currently, your Funding Statement reads as follows:

“Japan Society for the Promotion of Science”

Please include your amended statements within your cover letter; we will change the online submission form on your behalf.

My response:

I have revised the Acknowledgments section to remove the mention of the Japan Society for the Promotion of Science. The Funding Statement can remain as “Japan Society for the Promotion of Science.”

3. Thank you for stating the following in the Competing Interests section:

“I have read the journal's policy and the authors of this manuscript have the following competing interests: financial support was provided by Japan Society for the Promotion of Science, and data was provided by meisterstudio, Inc.”

Please confirm that this does not alter your adherence to all PLOS ONE policies on sharing data and materials, by including the following statement: "This does not alter our adherence to  PLOS ONE policies on sharing data and materials.” (as detailed online in our guide for authors http://journals.plos.org/plosone/s/competing-interests).  If there are restrictions on sharing of data and/or materials, please state these. Please note that we cannot proceed with consideration of your article until this information has been declared.

Please include your updated Competing Interests statement in your cover letter; we will change the online submission form on your behalf.

My response:

I have add following statements on my cover letter: “The data used in this study is third-party data, and I do not have the rights to share it, but researchers can obtain the data by applying for and receiving permission from the Informatics Research Data Repository of the National Institute of Informatics (<https://www.nii.ac.jp/dsc/idr/minhyo/>) by following the data availability statement.”

I additionally add following data availability statement on my cover letter.

“Data Availability Statement:

The employee review data necessary for reproducing this study was obtained from the “Minhyo Review Dataset" (<https://doi.org/10.32130/idr.16.1>), provided by meisterstudio, Inc. via the IDR Dataset Service of the National Institute of Informatics. To obtain the review data for reproducing this study, one first applies for and receives permission from the Informatics Research Data Repository of the National Institute of Informatics

(<https://www.nii.ac.jp/dsc/idr/minhyo/>). Then, anyone can access the data by processing the downloaded dataset according to the method indicated in the "Data collection" in the Methodology section of this paper.”

4. Please note that your Data Availability Statement is currently missing the DOI/accession number of each dataset OR a direct link to access each database. If your manuscript is accepted for publication, you will be asked to provide these details on a very short timeline. We therefore suggest that you provide this information now, though we will not hold up the peer review process if you are unable.

My response:

I have added the Data Availability Statement, which is mentioned in my response to the previous comment, to my cover letter. I followed the Data Availability Statement from another research paper (<https://doi.org/10.1371/journal.pone.0289368>) published in PLOS ONE, which also used data provided by the IDR Dataset Service of the National Institute of Informatics. I confirmed with the National Institute of Informatics before writing the Data Availability Statement.

5. Please amend either the title on the online submission form (via Edit Submission) or the title in the manuscript so that they are identical.

My response:

I have amended the manuscript title to match the online form as follows: "What Do Part-Time Employees in Japanese Chain Restaurants Talk About When Dissatisfied? Applying Structural Topic Modeling to Employee Reviews."

6. We notice that your supplementary tables are included in the manuscript file. Please remove them and upload them with the file type 'Supporting Information'. Please ensure that each Supporting Information file has a legend listed in the manuscript after the references list.

My response:

I have removed my supplementary table and uploaded with the file type “Supporting Information.” I have added S1 supporting table in the manuscript after the reference list.

7. Please review your reference list to ensure that it is complete and correct. If you have cited papers that have been retracted, please include the rationale for doing so in the manuscript text, or remove these references and replace them with relevant current references. Any changes to the reference list should be mentioned in the rebuttal letter that accompanies your revised manuscript. If you need to cite a retracted article, indicate the article’s retracted status in the References list and also include a citation and full reference for the retraction notice.

My response:

I have checked reference list. There is no retracted paper included in my list. There are no changes in my reference list.

**Comments to the Author**

Additional Editor Comments:
Technically, the article conforms in part. I suggest adding more information to the conclusion section or having the author discuss the research's limitations. Maybe it would be better to draw attention to the cultural traits as well. Aside from these recommendations, I think the data analysis and presentation in the manuscript are reasonable, and the statistical techniques employed are sufficient.

My response:

Thank you, dear editor. I have expanded the Discussion and Conclusion sections by adding the limitations including cultural traits and the practical implications of the findings for the chain restaurant industry in the last three paragraphs. Firstly, I addressed the limitation that extreme opinions are more likely to be collected in online reviews. Secondly, I added content focusing on Japanese work culture, backed by additional references. Finally, I concluded with future analyses for the chain restaurant industry based on the findings of this study. Below are the added paragraphs:

“However, the study has several limitations. The data used were derived from an online review platform that anyone could post. Previous research has shown that extreme opinions are more likely to be posted \cite{Hu2009-dd}, and this study found a particular abundance of reviews focusing on dissatisfaction. While the abundance of dissatisfaction reviews aligns with the primary objective of the study, to comprehensively understand what aspects of management and job content are appreciated by part-time employees, a different methodological approach is needed. Specifically, analyzing positive stories about employee satisfaction requires using data from platforms with some incentive to post reviews. For instance, on employee review sites used for job hunting, where users must post reviews to see others' reviews due to platform rules, there would be an incentive to post reviews even if they are not extreme opinions. Focusing on enhancing positive aspects, in addition to addressing dissatisfaction, is essential for improving the retention rate of part-time employees.

Another limitation is the reliance on data from a single Japanese platform, potentially limiting its global applicability. Japanese workplace culture is often characterized by long working hours, an emphasis on interpersonal relationships, and a high expectation of commitment to the company \cite{Aljayi2016-xd, Peltokorpi2013-tq, Zahari2020-ji}. Since this study focused on part-time workers with set working hours, there was no discussion of long working hours or overtime. However, many topics related to interpersonal relationships emerged, and the connection to relationship scores might reflect the influence of Japanese workplace culture. While studies using employee reviews from other countries \cite{Sainju2021-sh} also identified topics related to coworkers and supervisors, indicating that these are somewhat universal topics, comparative studies are needed to examine the relative frequency of these topics. Additionally, although not directly appearing in the topics, unique aspects of the Japanese service industry, such as 'Omotenashi' \cite{Miki2022-ph} and high service quality relative to cost \cite{Morikawa2016-xz}, might also affect the generalizability of the results. Further research should expand to diverse datasets and cross-cultural comparisons, aiding both Japanese and international companies in developing culturally informed management strategies.

This study analyzed the relationship between review topics and the satisfaction of part-time employees in the restaurant industry, which faces a labor shortage, as part of a multifaceted approach to reducing turnover rates. The analysis conducted in this study is expected to serve as a starting point for further empirical research on improving turnover rates by addressing the dissatisfaction mentioned in reviews. By combining review analysis with objective indicators such as profitability, company size, and turnover rate, it is possible to identify the types of companies and management systems that are more prone to specific dissatisfaction, which could result in more effective management policies tailored to the characteristics of each company’s part-time workforce. It is hoped that this study will inspire each company to address dissatisfaction from various angles and secure highly satisfied and stable part-time employees, thereby providing consistent service quality, and contributing to further improvement in the industry.”

Reviewer #1: From a technical point of view, the article partially complies. I recommend that the conclusion section be expanded, or the author should also address the limitations of the research. Perhaps it would be better to emphasize the cultural characteristics as well. Apart from these suggestions, I consider the statistical methods used in the manuscript to be adequate, and the analysis and presentation of the data are appropriate.

My response:

Thank you, dear reviewer. Similar to my response to the previous editor's comments, I have expanded the conclusion section and added limitations, particularly elaborating on cultural characteristics.

Sincerely yours

Hiroki Takahashi
University of Tsukuba
takahashi.hiroki.gn@u.tsukuba.ac.jp
